# Rapid and accurate prediction of cycloplegic refraction in Chinese children: development and validation of machine learning models

**DOI:** 10.7189/jogh.15.04281

**Published:** 2025-10-17

**Authors:** Yujia Liu, Jianmin Shang, Yuliang Wang, Xingxue Zhu, Chaoying Ye, Chongyang Wang, Xiaomei Qu

**Affiliations:** 1 Eye Institute and Department of Ophthalmology, Eye & ENT Hospital, Fudan University, Shanghai, China; 2 NHC Key laboratory of Myopia and Related Eye Diseases, Key Laboratory of Myopia and Related Eye Diseases, Chinese Academy of Medical Sciences, Shanghai, China; 3 Shanghai Research Center of Ophthalmology and Optometry, Shanghai, China; 4 Research and Development Department, Shanghai MediWorks, Shanghai, China

## Abstract

**Background:**

Uncorrected refractive error affects approximately 19 million children globally, resulting in preventable vision loss. However, cycloplegic refraction, the gold standard for assessment, remains largely inaccessible in low-resource settings. We aimed to develop and validate machine learning (ML) prediction models based on non-cycloplegic results to estimate the cycloplegic spherical equivalent (cSE).

**Methods:**

The internal dataset comprised refractive measurements and ocular biometric parameters collected from 3035 children’s eyes at the Eye & ENT Hospital of Fudan University, the research team’s primary hospital. The external validation sets consisted of 160 and 120 eyes, respectively, from two different centres. Based on ocular biometric parameters and non-cycloplegic spherical equivalent, we employed single and stacked ML models to predict cSE. We used regression metrics and agreement analysis between the predicted spherical equivalent (pSE) and cSE to assess prediction performances. We also created segmented models based on age and refractive groups. The generalisation performance of the models was assessed using evaluation metrics as well as correlation and agreement analyses in the external validations.

**Results:**

The stacked overall model outperformed single-algorithm models, achieving an *R*^2^ of 0.982 and a mean absolute error(MAE) of 0.360D. The MAE in segmented models ranged from 0.239D to 0.466D in the middle-aged groups and 0.226D to 0.420D in the high-aged groups, with better 95% limits of agreement between pSE and cSE than those in the overall model. External validation showed MAEs of 0.284D and 0.306D for the two datasets, with significant correlations, but lack of agreement between pSE and cSE.

**Conclusions:**

The ML models enable cSE prediction based on non-cycloplegic refraction data and ocular biometric parameters, providing a fast, practical method for estimating refractive error. Multicenter validation and targeted oversampling of rare refractive subgroups are required, however, before robust clinical implementation of the models.

Uncorrected refractive error and anisometropia can induce amblyopia, making refractive error one of the main causes of visual impairment [1–3]. One such impairment is myopia, the prevalence of which has been continuously rising worldwide in recent years, with an especially early onset and high incidence trend in China [4]. Importantly, both refractive error correction and the management of related complications increase the burden on the national economy [5].

Ametropia in children is difficult to identify [6], which is concerning, given that early diagnosis and intervention significantly reduce the incidence and severity of refractive errors and amblyopia [7]. Therefore, conducting refractive screenings and maintaining refractive development records has become a consensus in the academic community.

An inevitable problem in clinical practice is selecting a refraction measurement method. While cycloplegic refraction, for example, is the gold standard for children, it is impractical in school and community screening [8]. Non-cycloplegic refraction, meanwhile, often overestimates myopia and underestimates hyperopia, particularly in younger children with higher diopters [9]. Critically, both rapid and slow cycloplegia bear inherent clinical risks, including photophobia, impaired near vision, contraindications, and potential non-cooperation, thereby further challenging routine clinical application.

Cycloplegic spherical equivalent (cSE) has been observed to correlate with age, ocular biometric factors, and pre-cycloplegic refraction [5,10]. Thus, it is highly feasible to estimate the actual refractive error in follow-ups within a specific period using non-cycloplegic examination results, in addition to the required cycloplegic refraction at the initial visit.

Artificial intelligence (AI) is becoming increasingly utilised in medical research [11]. This includes machine learning (ML), a subfield of AI which uses algorithms to automatically recognise, classify, and predict new, unknown outcomes based on existing data. By generating features from existing variables, ML models often outperform traditional regression methods in predictive accuracy and stability [12,13].

With this in mind, we aimed to construct and validate ML prediction models based on non-cycloplegic refraction data and ocular biometric parameters to predict cSE.

## METHODS

### Study population

We included children aged 3–12 years meeting all of the following criteria: astigmatism not exceeding −2.00 D and best-corrected visual acuity greater than 0.3 in logarithm of the minimum angle of resolution (logMAR), where 0.0 represents standard 20/20 vision and higher values indicate worse acuity). We excluded those with severe ocular diseases, such as strabismus (except for intermittent exotropia), cataracts, glaucoma, retinopathy of prematurity, or previous intraocular surgery; past or current administration with atropine, orthokeratology lenses, or other myopia treatments; systemic diseases affecting refraction; and allergy or contraindications to cycloplegic.

We obtained the internal dataset from the Eye & ENT Hospital of Fudan University, which is our team’s primary research hospital, and the two external datasets from medical institutions in other districts of Shanghai, China. The underlying data were collected following the same standardised protocol and utilising the same equipment and measurement procedures, with the data collection supervised by a core team of investigators to ensure methodological uniformity.

The Ethics Committee of the Eye & ENT Hospital of Fudan University (Approval No. 2022015) in January 2022 approved the study, while we adhered to the Declaration of Helsinki in conducting the research. Written or spoken informed consent was obtained from the parents of the participants. Patients or the public were not involved in the design, or conduct, or reporting, or dissemination plans of our research.

### Sample size calculation

There is no established consensus on sample size determination for ML-based prediction models; it is recommended that they include at least 10 times the number of samples per predictor variable to reduce the risk of overfitting [14]. Using *R*, version 2.4.3 (R Core Team, Vienna, Austria) and the ‘pmsampsize’ package, we set the adjusted *R*^2^ to 0.95 with an acceptable difference of 0.05. Assuming the multiplicative margin of error for estimating the intercept and a residual standard deviation less than 1.1, and considering the average and standard deviation of the outcome variable from the pilot experiment, the calculated sample size was determined to be 247 cases. To prevent overfitting in the ML models (particularly when *R*^2^ value is excessively high), the sample size was increased 10 times, resulting in a final target sample size of 2500 cases for the internal dataset.

We determined the minimum sample size for each external centre using the calculation method proposed by Archer and colleagues [15], which sets *R*^2^ to 0.93 and the standard error of *R̂*^2^_val_ to 0.0255 to achieve a 95% confidence interval. Finally, we set the sample size for each external center to 100. This was done per the following formula:



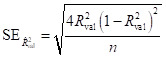





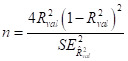



### Measurements

We performed slit-lamp and ophthalmoscopic examinations to assess the anterior segments and fundus of the eyes, and cover tests were conducted to evaluate eye alignment. Basic information was collected from electronic medical records at the outpatient clinic.

Non-cycloplegic refraction, including non-cycloplegic spherical diopter (nS) and spherical equivalent (nSE), was measured using an autorefractor (ARK 510A, NIDEK, Japan), which represents an essential component of routine clinical practice. Ocular parameters such as axial length (AL), mean keratometry (K), flat keratometry (K1), steep keratometry (K2), corneal astigmatism (δK), anterior chamber depth (ACD), aqueous depth (AQD), lens thickness (LT), central corneal thickness (CCT), and white-to-white distance (WTW) were assessed with the IOL Master 700 (Carl Zeiss, Jena, Germany). The corneal radius (CR, in mm) was determined from mean keratometry values (in diopters) using the established conversion factor of 337.5 divided by K.

Cycloplegic autorefraction was performed again after sufficient cycloplegia with tropicamide 0.5% (Bausch & Lomb Pharmaceutical Co., Ltd, Shandong, China), administered at five-minute intervals for a total of five doses. Astigmatism was analysed as a vector using the Cravy method, where J_0_ = (−δK/2) cos(2 × axis) represents horizontal and vertical astigmatism, and J_45_ = (−δK/2) sin(2 × axis) represents oblique astigmatism at 45°, with ‘axis’ being the axis of astigmatism. Spherical equivalent (SE) was calculated as the sphere plus half of the cylinder.

### Machine learning

#### Dataset splitting

**Figure 1:**
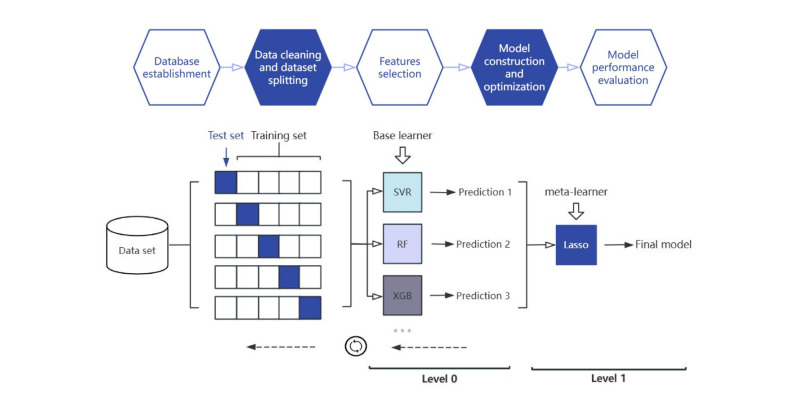
Process of establishing the predictive model diagram of the 5-fold stacking model.

We first excluded individuals with missing values after determining that these values were missing at random. Then, we used a five-fold cross-validation method in which the complete data were randomly divided into five equal-sized subsets. We performed five rounds of training and testing, using four subsets as the training set and the remaining subset as the testing set (**Figure 1**).

#### Features selection

We initially selected candidate variables for predicting cSE based on published literature and preliminary work, after which we used decision tree models to heuristically search for feature (*i.e.* predictor variable) subsets in the training set and output the feature importance (*i.e.* quantitative measure of each variable's predictive contribution). We then ranked features in descending order and determined the threshold based on the importance of feature selection. To further guard against overfitting and reduce dimensionality, we constructed new features (*i.e.* clinically interpretable combinations, *e.g.* axial length-to-corneal curvature ratio (AL/CR)) based on the original ones.

To address interpretability and multicollinearity concerns, we performed correlation analyses between the top-ranked features and the target variable cSE, and calculated correlation coefficients and their significance (**Online Supplementary Document**). We applied Pearson’s linear correlation if both the feature and the target variable were normally distributed, and Spearman’ rank correlation if at least one variable was skewed or ordinal.

#### Overall model construction and hyperparameter optimisation

We used various algorithms, including least absolute shrinkage and selection operator (LASSO) regression, support vector regression (SVR), random forest (RF), and extreme gradient boosting (XGBoost), to build the prediction models. The stacking structure consisted of two layers of algorithms. Level 0 (base learners) included algorithms such as SVR and XGBoost, selected for their ability to capture complex, nonlinear relationships in refractive data. Level 1 (meta-learner) used LASSO regression for its interpretability and L1 regularisation, which reduces overfitting by assigning optimal weights to base-model predictions. Algorithmic diversity was maintained by combining distinct learning paradigms: kernel methods (SVR), tree ensembles (XGBoost, RF), and regularised linear regression (LASSO). Additionally, we ran Bayesian optimisation (*via* ‘scikit-optimize’, version 0.9.0) for 50 iterations per model to determine the best hyperparameters for each model during the machine-learning process.

#### Model performance evaluation

We used several metrics used to assess model performance: goodness of fit (*R*^2^), mean error (ME), mean absolute error (MAE), median error (MedE), median absolute error (MedAE), root mean square error (RMSE), as well as the proportion of predictions within ±0.25D, ±0.50D, ±0.75D, and ±1.00D of the actual values. This calculation was done per the formulas:



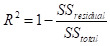





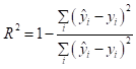





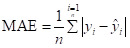





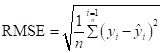



We generated Bland-Altman plots using MedCalc, version 22.021 (MedCalc Software, Ostend, Belgium), to analyse the agreement between the predicted spherical equivalent (pSE) and cSE.

#### Construction and evaluation of segmented models

We divided participants into 18 subgroups according to age and refractive status. We repeated the same modelling process for each subgroup, and selected the best one to form the final segmented models.

The age groups were formed as follows:

– 3–6 years (preschool stage) for low-aged group;

– 7–9 years (early school stage) for middle-aged group;

– 10–12 years (pre-adolescence stage) for high-aged group.

The categorisation for refractive status was done according to the non-cycloplegic spherical equivalent (nSE), as follows:

– SE<−6.00 D for high myopia,

– −6.00 D ≤ SE < −3.00 D for moderate myopia;

– −3.00 D ≤ SE ≤ 0.00 D for low myopia;

– 0.00 D < SE ≤ 3.00 D for low hyperopia;

– 3.00 D < SE ≤ 6.00 D for moderate hyperopia;

– SE > 6.00 D for high hyperopia.

### Statistical analysis

We used the Shapiro-Wilk test to assess the normality of continuous variables, presenting those with a normal distribution as means and standard deviations, and those with a skewed distribution as medians and interquartile ranges. We used the χ^2^ test or Fisher’s exact test for comparing categorical variables, the Kruskal-Wallis test for comparing variables with skewed data, and one-way ANOVA or Welch’s ANOVA for normally distributed data with or data without equal variance. We applied the Bonferroni correction for multiple pairwise comparisons.

We performed a correlation analysis to calculate the correlation coefficients for the predicted values of pSE and cSE. Bland-Altman plots were generated for agreement analysis.

We performed the analyses using SPSS, version 26.0 (IBM Corp., Armonk, New York, USA), MedCalc, 22.021 (MedCalc Software, Ostend, Belgium), and Microsoft Excel 2019 (Microsoft Excel, Redmond, Washington, USA). A significance level of α = 0.05 was set for type I error.

## RESULTS

### Performance of the overall model

**Figure 2:**
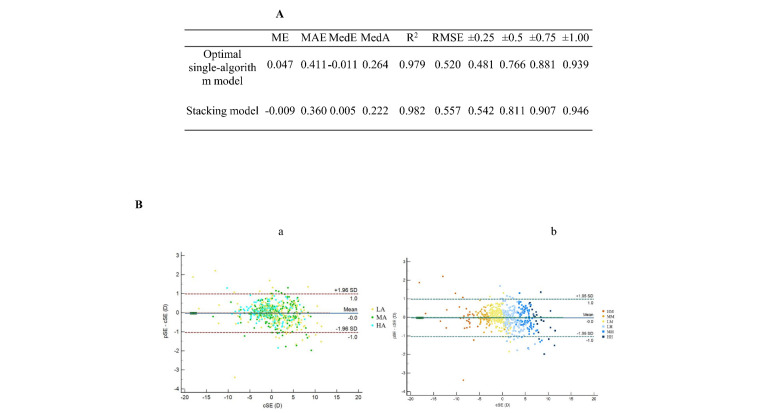
Predictive performance and agreement analysis of pSE and cSE for the overall model. **Panel A.** Predictive performance of the overall model. **Panel B.** Agreement analysis of pSE and cSE for the overall model, categorised by age (a) and refractive status (b). HA – high-aged group, HH – high hyperopia group, HM – high myopia group, LA – low-aged group, LH – low hyperopia group, LM – low myopia group, MA – middle-aged group, MH – middle hyperopia group, MM – middle myopia group, nSE – non-cycloplegic spherical equivalent, pSE – predictive spherical equivalent.

The internal dataset included refractive measurements and ocular biometric parameters from 3035 eyes (1503 male, 1532 female), while external datasets 1 and 2 consisted of 160 (76 male, 84 female) and 112 (36 male, 76 female) eyes, respectively (**Table 1**). The final selected features included age, nSE, nS, AL, AL/CR, K, K1, δK, AQD, ACD, LT, CCT, and WTW. The SVR algorithm exhibited the best performance among the individual models, with an *R*^2^ of 0.979 and MAE of 0.411 diopters (D) (**Figure 2**, Panel A). The prediction error was within ±0.25D for 48.1% of the cases and within ±0.50D for 76.6% of the cases. The stacking ensemble approach achieved superior performance, with an *R*^2^ of 0.982 and MAE of 0.360 D, outperforming LASSO and other base models. The prediction error was within ±0.25D for 54.2% of the cases and within ±0.50D for 81.1% of the cases. The 95% limits of agreement (LoA) between pSE and cSE of the overall model was −1.01D to 1.03D (*P* = 0.680), indicating agreement between pSE and cSE (**Figure 2**, Panel B).

**Table 1:** Comparison of characteristics between datasets

	Internal dataset (n = 3035)	External dataset 1 (n = 160)	External dataset 2 (n = 112)	*P*-value (*P*_I-E1_, *P*_I-E2_, *P*_E1-E2_)
**Gender**				
**Female, n (%)**	1532 (50.5)	84 (52.5)	76 (67.9)	0.003 (0.646, 0.001, 0.011)
**Male, m (%)**	1503 (49.5)	76 (47.5)	36 (32.1)	
**Age in years**				<0.001 (<0.001, 0.003, 0.790)
MD (IQR)	8 (3)	9 (3)	8.50 (3)	
Range	3, 12	5, 12	4, 12	
**nS(D)**				<0.001 (<0.001, <0.001, 0.001)
MD (IQR)	0.00 (4.25)	−1.87 (1.25)	−1.12 (1.22)	
Range	−19.50, 15.25	−8.25, 1.50	−4.25, 4.12	
**nSE(D)**				<0.001 (<0.001, 0.001, 0.001)
MD (IQR)	−0.37 (4.13)	−2.12 (1.25)	−1.34 (1.23)	
Range	−21.38, 14.63	−8.87, 1.25	−4.50, 3.69	
**cS (D)**				<0.001 (<0.001, <0.001, 0.003)
MD (IQR)	0.50 (5.00)	−1.50 (1.25)	−0.87 (1.13)	
Range	−19.00, 16.00	−7.75, 1.75	−4.12, 4.12	
**cSE (D)**				<0.001 (<0.001, <0.001, 0.002)
MD (IQR)	0.25 (5.00)	−1.87 (1.50)	−1.05 (1.23)	
Range	−20.75, 15.38	−8.50, 1.50	−4.37, 3.69	
**δSE(D)**				<0.001 (<0.001, <0.001, 0.361)
MD (IQR)	0.38 (0.88)	0.25 (0.38)	0.25 (0.44)	
Range	−6.00, 8.88	−0.50, 3.00	−4.49, 1.99	
**J_0_**				0.629
MD (IQR)	0.00 (0.40)	0.00 (0.26)	0.00 (0.21)	
Range	−2.62, 2.47	−0.84, 1.24	−0.64, 0.37	
**J_45_**				0.091
MD (IQR)	0.00 (0.41)	0.00 (0.23)	0.04 (0.19)	
Range	−2.84, 2.11	−0.84, 1.26	−0.48, 0.80	
**AL in mm**				<0.001 (<0.001, 0.051, <0.001)
MD (IQR)	23.24 (2.26)	24.31 (0.98)	23.53 (1.28)	
Range	15.98, 33.43	22.93, 26.78	20.99, 25.78	
**K (D)**				0.006 (0.637, 0.012, 0.006)
MD (IQR)	43.10 (2.01)	43.02 (1.72)	43.36 (1.98)	
Range	37.57, 49.28	40.21, 46.20	40.29, 46.88	
**K1 (D)**				0.001 (1.000, <0.001, 0.018)
MD (IQR)	42.38 (1.94)	42.52 (1.76)	42.68 (2.05)	
Range	36.63, 49.95	39.60, 45.65	39.59, 46.69	
**K2 (D)**				0.002 (0.009, 0.210, 0.002)
MD (IQR)	43.82 (2.20)	43.56 (1.73)	44.15 (2.12)	
Range	38.15, 50.48	23.80, 46.91	40.70, 47.99	
**δK (D)**				<0.001 (<0.001, <0.001, 0.674)
MD (IQR)	−1.43 (0.90)	−1.06 (0.67)	−1.17 (0.70)	
Range	−5.65, 1.46	−2.83, 1.68	−3.77, −0.27	
**ACD in mm**				<0.001 (<0.001, 0.001, 0.057)
MD (IQR)	3.57 (0.40)	3.73 (0.29)	3.63 (0.28)	
Range	2.29, 4.66	3.22, 4.10	3.24, 4.19	
**AQD in mm**				<0.001 (<0.001, <0.001, 0.041)
MD (IQR)	3.02 (0.40)	3.19 (0.30)	3.08 (0.27)	
Range	2.08, 4.09	2.69, 3.56	2.66, 3.65	
**LT in mm**				<0.001 (<0.001, <0.001, 0.531)
MD (IQR)	3.47 (0.28)	3.35 (0.17)	3.37 (0.19)	
Range	2.84, 4.35	3.04, 3.82	3.07, 3.69	
**CCT in μm**				0.915
MD (IQR)	549.00 (44.00)	550.00 (43.50)	550.00 (33.75)	
Range	442.00, 669.00	460.00, 626.00	501.00, 650.00	
**WTW in mm**				0.001 (0.001, 1.000, 0.005)
MD (IQR)	12.20 (0.50)	12.30 (0.57)	12.10 (0.54)	
Range	10.10, 13.70	11.40, 13.10	11.23, 13.17	

ACD – anterior chamber depth, AL – axial length, AQD – aqueous depth, CCT – central corneal thickness, cS – cycloplegic spherical diopter, cSE – cycloplegic spherical equivalent, D – diopter, E1 – external dataset 1, E2 – external dataset 2, I – internal dataset, IQR – interquartile range, K – mean keratometry, K1 – flat keratometry, K2 – steep keratometry, LT – lens thickness, MD – median, nS – non-cycloplegic spherical diopter, nSE – non-cycloplegic spherical equivalent, WTW – white-to-white, δK – difference between K1 and K2

### Performance of the segmented model

Sample sizes and predictive performance of the stacking models for each age group are detailed in the **Online Supplementary Document**. Except for the groups with very few cases of high refractive errors, the MAE ranged from 0.222 to 0.492D in the low-aged group, from 0.239 to 0.466D in the middle-aged group, and from 0.226 to 0.420D in the high-aged group (**Online Supplementary Document**). Bland-Altman plots comparing pSE *vs*. cSE across different age groups demonstrated clinically acceptable agreement between predicted and cycloplegic spherical equivalent values. The 95% LoA were −1.04D to 0.98D (*P* = 0.188) for the whole, with −1.31D to 1.15D (*P* = 0.066) for the low-aged group, −0.95D to 0.90D (*P* = 0.349) for the middle-aged group, and −0.76D to 0.85D (*P* = 0.186) for the high-aged group, indicating agreement between pSE and cSE (**Online Supplementary Document**).

### External validation

**Figure 3:**
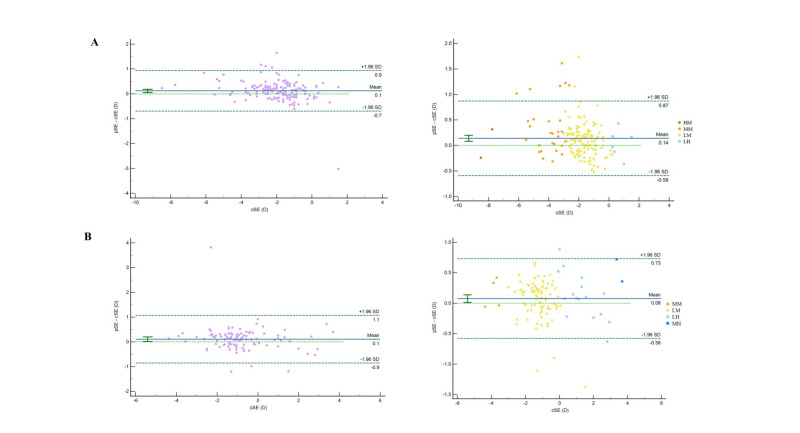
Agreement analysis of pSE and cSE for the overall model and segmented model in external dataset 1 (**Panel A**) and external dataset 2 (**Panel B**). HA – high-aged group, HH – high hyperopia group, HM – high myopia group, LA – low-aged group, LH – low hyperopia group, LM – low myopia group, MA – middle-aged group, MH – middle hyperopia group, MM – middle myopia group, nSE – non-cycloplegic spherical equivalent, pSE – predictive spherical equivalent.

We observed differences in age, nSE, cSE, and ocular biometric parameters were observed among the three datasets (**Table 1**). The correlation coefficient between the pSE and cSE of the segmented model was 0.966 (*P* < 0.001) for external dataset 1 and 0.973 (*P* < 0.001) for external dataset 2. In both external datasets, the segmented model showed a higher correlation between pSE and cSE than the overall model. According to the Bland-Altman plot, the 95% LoA were −0.591D to 0.872D in external dataset 1 (*P* < 0.001) and −0.579D to 0.729D in external dataset 2 (*P* = 0.019), indicating a statistical difference between pSE and cSE, suggesting a lack of agreement (**Figure 3**).

## DISCUSSION

Accurate diopter measurements without cycloplegia are critical for children’s refractive screening and follow-up. Previous studies have attempted to predict refractive status or specific cSE values in children. One such study reported a classification accuracy of only 62% without cycloplegia [16]. Sankaridurg and colleagues [16] established a multiple regression model for Chinese children aged 4–15 years, but their model misclassified 23% of eyes during validation. Magome and colleagues constructed a prediction model for Japanese children aged 2–9 years with high correlation to actual measurements, but did not perform agreement tests [17]. Wang and colleagues, meanwhile, predicted cSE with a mean error of 0.06D, significantly lower than the average standard error between nSE and cSE [18]. Another school-based study employed six single-algorithm models to predict cSE and myopia status in Chinese students, achieving high *R*^2^ values (from 0.913 to 0.935) and low MAE (0.393–0.480 D) [19]. However, this study lacked data on preschool children, which is a critical period of refractive development.

Here we used a decision tree model to extract top-ranked important features. Notably, we employed ML techniques to create the AL/CR parameter, an indicator of myopia onset and progression [20–22], which we calculated from AL and corneal curvature, then standardised by Z-score before model training. Unlike isolated AL or K measurements, AL/CR captures compensatory interactions between axial elongation and corneal curvature changes during refractive development. Corneal curvature stabilises with age, while AL continues to increase, raising the AL/CR ratio. Relatedly, He and colleagues found the AL/CR ratio had an area under the receiver operating characteristic curve (AUC) of 0.910, outperforming AL alone (AUC = 0.822) [20].

While prediction using single ML models has inherent limitations, ensemble methods generally achieve enhanced accuracy and stability [19,23,24]. Specifically, stacking approaches further reinforce these advantages by integrating base learners' outputs to train meta-learners [25]. In our study, the stacking model improved *R*^2^ from 0.979 to 0.982, reduced MAE from 0.411D to 0.360D, and increased prediction accuracy within ±0.25D from 48.1% to 54.2%, and within ±0.50D from 76.6% to 81.1%. This magnitude of error is clinically acceptable and unlikely to alter management decisions for cycloplegic referral or myopia control. However, the lower proportion of predictions within ±0.25D suggests risks in precision-sensitive scenarios, particularly for borderline myopia. Prediction errors exceeding ±0.25D could trigger unnecessary interventions or delay corrective treatment, increasing risks of amblyopia development and accelerated axial elongation. Our future work will formalise predictive-value analyses at clinically relevant thresholds (±0.25D, ±0.50D) and simulate the model’s impact on downstream patient outcomes.

The segmented model in our study performed better in the agreement analysis than the overall model. However, constructing it was challenging, due to the limited number of cases in certain age and refractive groups, affecting prediction accuracy for specific populations, such as older children with hyperopia and younger children with moderate-to-high myopia. This condition may stem from the varying distributions of refractive errors by age. Typically, refractive development progresses from hyperopia at birth toward emmetropia as AL increases. Consequently, myopia is less common in younger children (ages 3–6), while moderate-to-high hyperopia is rare in older children (ages 10–12). Limited data for myopia groups due to growing awareness of myopia prevention, and the difficulty in collecting reliable examination data from young children, also contributed to this challenge. Notably, systematic errors were largest in extreme refractive subgroups and eyes with AL >25 mm, likely due to limited training data and nonlinear biometric interactions. Therefore, targeted sampling and domain-specific constraints should be used to reduce these biases.

It should be noted that, although we did not perform heteroskedasticity tests, inspection of the 95% LoA and Bland-Altman analyses across all subgroups revealed no funnel-shaped or progressively widening variance with rising cSE (**Figure 3**, Panels B–D). This supports the view that prediction-error variance remains approximately stable across age groups and refractive error ranges. In subsequent studies, formal heteroskedasticity tests alongside global sensitivity analyses are recommended to rigorously quantify how uncertainty in biometric measurements propagates into prediction error.

Most clinical studies on predictive models lack external validation, while models in studies that have it perform worse than in internal validation. Here, the overall model performed reasonably well in the two external datasets, with MAEs of 0.284D and 0.306D and prediction accuracy within ±0.25D of 59.4% and 54.5%, respectively. While the segmented model outperformed the overall model in some subgroups, it struggled in others, possibly due to small sample sizes or cohort differences. Notably, the segmented model showed stronger correlations between pSE and cSE than the overall model (0.966 *vs*. 0.959 in external dataset 1; 0.973 *vs*. 0.942 in external dataset 2). This suggests that larger external validation samples covering diverse ages and refractive statuses could enhance the segmented model’s generalisability, indicating the need for further refinement.

This study has several limitations. First, all datasets included only children from Shanghai, China, restricting the geographic generalisability of the results. Second, 0.5% tropicamide for cycloplegia, which aligns with the routine clinical practice in China, may yield different cSE values compared to cyclopentolate used elsewhere. To address potential errors from residual accommodation, future studies should conduct comparative trials involving multiple cycloplegic agents, including cyclopentolate, the international gold-standard cycloplegic. Finally, the external validation dataset lacked a broad age range and refractive error distribution, which limited the evaluation of certain segments within the stacked model. Children with strabismus, high astigmatism, and prior myopia treatment should also be considered and included in specific subgroups.

## CONCLUSIONS

This study introduces a segmented stacking modelling approach for predicting cycloplegic spherical equivalents, specifically tailored to paediatric refractive error assessment. In the future, combining vision screening devices to collect data and develop ML models could significantly enhance the accuracy and efficiency of refractive screening, particularly in young children with low compliance. Furthermore, the model developed here can be refined by incorporating additional predictor variables or adjusting algorithms and hyperparameters to better adapt to diverse populations. Future multicentre studies with prospective designs should recruit enough participants per centre to adequately power subgroup analyses, intentionally oversample rare refractive categories (*e.g.* high hyperopia in older and high myopia in younger children), and incorporate geographically diverse populations to validate transferability across ethnic groups. This could lead to the improvement and broader applicability of the predictive model for refractive error assessment.

The ML models we established here enable cSE prediction based on non-cycloplegic refraction data and ocular biometric parameters, offering a practical solution for estimating refractive error rapidly and accurately.

## Additional material


Online Supplementary Document
