# Supplementary material for: Rapid and accurate prediction of cycloplegic refraction in Chinese children: development and validation of machine learning models
Source: J Glob Health. 2025 Oct 17;15:04281. doi: 10.7189/jogh.15.04281 (PMC12532443; doi:10.7189/jogh.15.04281)
Supplement: Online Supplementary Document [file jogh-15-04281-s001.pdf]

Supplement to: Liu Y, Shang J, Wang Y, Zhu X, Ye C, Wang C, Qu X. Rapid and accurate prediction of cycloplegic refraction in Chinese children: development and validation of machine learning models. J Glob Health. 2025;15:04281.

Table 1. Top features correlated with cSE: correlation coefficients and significance

	Age	nSE	nS	AL	K	K_1	δK	ACD	AQD	LT	CCT	WTW
Correlation coefficients	-0.256	0.973	0.979	-0.871	-0.176	-0.200	-0.120	-0.560	-0.572	0.371	0.089	-0.061
P	<0.001	<0.001	<0.001	<0.001	<0.001	<0.001	<0.001	<0.001	<0.001	<0.001	0.001	0.017

nSE, non-cycloplegic spherical equivalent; nS, non-cycloplegic spherical diopter; AL, axial length; K, mean keratometry; K_1, flat keratometry; δK, corneal astigmatism; ACD, anterior chamber depth; AQD, aqueous depth; LT, lens thickness; CCT, central corneal thickness; WTW, white-to-white distance
